# Supplementary material for: Serum microRNA Profiles and Pathways in Hepatitis B-Associated Hepatocellular Carcinoma: A South African Study
Source: Int J Mol Sci. 2024 Jan 12;25(2):975. doi: 10.3390/ijms25020975 (PMC10815595; doi:10.3390/ijms25020975)
Supplement: Supplementary file 1 [file ijms-25-00975-s001.zip › Supplementary Table S1.pdf]

Supplementary Table S1: HCC serum miRNA Characterized/mappable UMI >1

miRNA	Base mean	log2FC	Std error	statistic	p-value	p-adj
hsa-miR-373-3p	5.719696	8.42231	0.883551	6.14653	7.92E-10	6.81E-09
hsa-miR-122-3p	15.14987	6.65198	0.732586	9.509009	1.92E-21	5.55E-20
hsa-miR-3591-5p	10.46715	6.466475	0.76204	8.54514	1.28E-17	2.33E-16
hsa-miR-1290	9.37994	6.375868	0.760523	7.381123	1.57E-13	1.83E-12
hsa-miR-665	20.96892	6.247295	0.939978	23.91597	0	1E-123
hsa-miR-4488	29.87861	5.619053	0.581307	9.767734	1.55E-22	5.06E-21
hsa-miR-4492	5.310284	5.528107	0.796688	6.995202	2.65E-12	2.84E-11
hsa-miR-296-3p	16.21069	5.480001	0.599385	9.074058	1.15E-19	2.68E-18
hsa-miR-127-5p	14.3652	5.291995	1.015118	5.260875	1.43E-07	8.78E-07
hsa-miR-4532	3698.692	5.142918	0.335717	15.32154	0	5.38E-51
hsa-miR-483-5p	44961.15	4.885663	0.323756	15.09255	0	1.48E-49
hsa-miR-455-5p	2.782882	4.60887	0.861528	5.399754	6.67E-08	4.3E-07
hsa-miR-6087	298.347	4.605506	0.283789	16.22821	0	7.81E-57
hsa-miR-7704	7.803728	4.503588	0.824031	5.452906	4.96E-08	3.28E-07
hsa-miR-1246	693.339	4.428349	0.282388	15.6819	0	2.46E-53
hsa-miR-4497	2.765307	4.312048	1.147763	3.466027	0.000528	0.001823
hsa-miR-1247-5p	15.98426	4.226732	0.785575	5.372836	7.75E-08	4.93E-07
hsa-miR-3194-5p	2.340388	4.091444	1.088568	3.658828	0.000253	0.000955
hsa-miR-412-5p	3.453552	4.070963	1.091257	3.787424	0.000152	0.000606
hsa-miR-512-3p	4.751975	4.070027	1.272652	3.102243	0.001921	0.005738
hsa-miR-433-3p	2.734157	4.068241	1.195951	3.181692	0.001464	0.004456
hsa-miR-483-3p	570.5936	3.935395	0.302586	13.00646	1.12E-38	6.31E-37
hsa-miR-494-3p	102.4987	3.808545	0.427589	8.907558	5.22E-19	1.07E-17
hsa-miR-320c	3978.861	3.708797	0.234175	15.83808	0	2.78E-54
hsa-miR-3180-3p	4.47244	3.432165	1.298102	2.876611	0.00402	0.01059
hsa-miR-887-3p	1.510252	3.269586	1.069255	3.065509	0.002173	0.006376
hsa-miR-323a-3p	22.22943	3.233736	0.509258	4.612819	3.97E-06	1.95E-05
hsa-miR-4516	138.9756	3.165472	0.242077	13.07597	0	3.16E-37
hsa-miR-452-5p	27.88144	3.108166	0.421811	7.36337	1.79E-13	2.04E-12
hsa-miR-663a	1.563521	3.049184	1.221206	2.612142	0.008998	0.021826
hsa-miR-3180	3.461276	2.998256	1.314113	2.571372	0.01013	0.024212
hsa-miR-365a-3p	2.806654	2.933892	0.80204	3.629171	0.000284	0.001055
hsa-miR-9-3p	21.01147	2.829662	0.49781	5.681111	1.34E-08	1.06E-07
hsa-miR-885-5p	17.12898	2.806151	0.536965	5.222057	1.77E-07	1.06E-06
hsa-miR-885-3p	17.9859	2.750249	0.568132	4.839828	1.3E-06	6.77E-06
hsa-miR-34a-5p	132.7026	2.710635	0.208432	13.00413	1.16E-38	6.31E-37
hsa-miR-1303	1.193861	2.681309	1.018455	2.605043	0.009186	0.022174
hsa-miR-429	7.153858	2.67148	0.716214	3.725674	0.000195	0.000746
hsa-miR-136-3p	12.22469	2.617716	0.662839	3.947409	7.9E-05	0.000337

hsa-miR-130b-3p	344.0793	2.579866	0.209296	12.32628	6.54E-35	2.91E-33
hsa-miR-192-5p	2452.268	2.537629	0.202326	12.54233	4.38E-36	2.15E-34
hsa-miR-320d	395.0843	2.508955	0.220255	11.39128	4.62E-30	1.89E-28
hsa-miR-210-3p	49.77771	2.449662	0.328359	7.459668	8.67E-14	1.06E-12
hsa-miR-218-5p	1.089152	2.441803	1.002239	2.398495	0.016463	0.037174
hsa-miR-874-3p	37.20805	2.369551	0.338818	6.991942	2.71E-12	2.84E-11
hsa-miR-122-5p	65595.74	2.135302	0.229412	9.307842	1.3E-20	3.36E-19
hsa-miR-365b-3p	5.442416	2.100319	0.62992	3.326733	0.000879	0.002851
hsa-miR-200b-3p	11.29843	2.0396	0.568542	3.585751	0.000336	0.001229
hsa-miR-320b	1244.831	2.018994	0.212063	9.52082	1.72E-21	5.26E-20
hsa-miR-625-3p	154.9778	-0.50358	0.145735	-3.45542	0.000549	0.001844
hsa-miR-15a-5p	583.2931	-0.51479	0.136678	-3.76647	0.000166	0.000654
hsa-miR-140-3p	710.4365	-0.53017	0.114606	-4.626	3.73E-06	1.86E-05
hsa-miR-16-5p	57609.45	-0.54342	0.144709	-3.75528	0.000173	0.000673
hsa-miR-301a-3p	5.980979	-0.54993	0.451827	-3.08362	0.002045	0.006073
hsa-miR-664a-5p	76.86328	-0.55188	0.16317	-3.38219	0.000719	0.002381
hsa-miR-363-3p	152.478	-0.56381	0.148664	-3.79246	0.000149	0.000599
hsa-miR-26b-5p	3497.34	-0.56674	0.101464	-5.58564	2.33E-08	1.7E-07
hsa-miR-191-5p	2691.166	-0.56905	0.094282	-6.03567	1.58E-09	1.31E-08
hsa-miR-125a-5p	896.9981	-0.57093	0.114116	-5.00304	5.64E-07	3.07E-06
hsa-let-7g-5p	1025.99	-0.57368	0.091575	-8.71229	2.98E-18	5.61E-17
hsa-miR-223-5p	203.6451	-0.58666	0.134846	-4.35059	1.36E-05	6.34E-05
hsa-miR-145-5p	23.97402	-0.59778	0.253434	-2.35847	0.01835	0.040871
hsa-miR-126-3p	9528.341	-0.60831	0.107886	-5.63846	1.72E-08	1.31E-07
hsa-miR-181a-2-3p	26.29231	-0.61605	0.223112	-2.76093	0.005764	0.014558
hsa-miR-106b-3p	129.9557	-0.62932	0.153457	-4.10091	4.12E-05	0.000182
hsa-miR-199b-3p	249.7963	-0.66604	0.105225	-6.32965	2.46E-10	2.23E-09
hsa-miR-17-5p	36.72098	-0.67313	0.204681	-4.75617	1.97E-06	9.97E-06
hsa-miR-199a-3p	2657.206	-0.67339	0.097297	-6.92098	4.49E-12	4.4E-11
hsa-miR-2110	15.32545	-0.67786	0.259293	-2.61384	0.008953	0.021826
hsa-miR-339-5p	138.1395	-0.6925	0.124057	-5.58213	2.38E-08	1.71E-07
hsa-miR-584-5p	404.9096	-0.6978	0.131478	-5.30734	1.11E-07	6.9E-07
hsa-miR-151a-3p	1234.377	-0.73369	0.110309	-6.6512	2.91E-11	2.79E-10
hsa-miR-409-3p	129.1007	-0.77119	0.211625	-3.64405	0.000268	0.001004
hsa-miR-324-3p	16.12407	-0.78378	0.261078	-3.00179	0.002684	0.007558
hsa-miR-182-5p	273.3272	-0.81254	0.169072	-4.80583	1.54E-06	7.95E-06
hsa-miR-190a-5p	187.2302	-0.82153	0.153502	-5.35187	8.7E-08	5.47E-07
hsa-miR-454-3p	96.08897	-0.8852	0.159384	-5.55372	2.8E-08	1.93E-07
hsa-let-7b-5p	17716.19	-0.88964	0.155153	-5.73395	9.81E-09	7.88E-08
hsa-miR-146a-5p	6559.699	-0.90415	0.107412	-8.41761	3.84E-17	6.72E-16
hsa-let-7i-5p	9798.911	-0.92264	0.112125	-8.22864	1.89E-16	3.2E-15

hsa-miR-25-5p	6.84924	-0.95195	0.417757	-2.27718	0.022775	0.049599
hsa-miR-181a-3p	12.89554	-0.96086	0.289184	-3.32203	0.000894	0.002881
hsa-let-7d-5p	902.5911	-0.97507	0.1274	-7.65358	1.95E-14	2.9E-13
hsa-miR-1277-5p	30.42358	-0.98986	0.253285	-3.90761	9.32E-05	0.000387
hsa-miR-3138	5.839791	-1.00125	0.405343	-2.46846	0.01357	0.03107
hsa-miR-744-5p	221.4047	-1.02964	0.116393	-8.84621	9.05E-19	1.77E-17
hsa-miR-139-3p	53.08787	-1.03265	0.187239	-5.5151	3.49E-08	2.37E-07
hsa-miR-425-5p	1124.783	-1.04312	0.106034	-9.83762	7.75E-23	2.71E-21
hsa-miR-140-5p	35.30478	-1.0457	0.200346	-5.21905	1.8E-07	1.06E-06
hsa-miR-16-2-3p	9.724804	-1.05514	0.413007	-2.55315	0.010675	0.025149
hsa-miR-191-3p	6.593383	-1.07549	0.410798	-2.61571	0.008904	0.021815
hsa-miR-155-5p	161.2596	-1.129	0.147754	-7.64098	2.16E-14	3E-13
hsa-miR-576-5p	23.6236	-1.14706	0.279315	-4.1059	4.03E-05	0.000179
hsa-let-7a-5p	13460.63	-1.15588	0.129421	-8.93112	4.22E-19	8.98E-18
hsa-miR-485-3p	13.92268	-1.15618	0.448428	-2.577	0.009966	0.023938
hsa-miR-196b-5p	61.10851	-1.19579	0.218811	-5.46457	4.64E-08	3.11E-07
hsa-miR-590-3p	10.71471	-1.21073	0.395937	-3.05615	0.002242	0.006462
hsa-miR-1273h-3p	8.081063	-1.21756	0.366892	-3.31737	0.000909	0.00291
hsa-miR-628-3p	22.36768	-1.21775	0.286407	-4.2509	2.13E-05	9.66E-05
hsa-miR-671-3p	6.135155	-1.21877	0.401113	-3.03649	0.002393	0.006779
hsa-miR-93-3p	8.556188	-1.23017	0.41457	-2.96542	0.003023	0.008321
hsa-let-7f-5p	13136.22	-1.23639	0.131157	-9.42677	4.23E-21	1.15E-19
hsa-miR-98-5p	157.1162	-1.25714	0.180782	-6.95381	3.56E-12	3.56E-11
hsa-miR-142-3p	7768.964	-1.27476	0.142683	-8.9342	4.1E-19	8.98E-18
hsa-miR-139-5p	8.746202	-1.28118	0.411129	-3.11468	0.001841	0.00557
hsa-miR-625-5p	16.94864	-1.29471	0.295801	-4.37571	1.21E-05	0.000057
hsa-miR-103b	115.6996	-1.30687	0.186929	-6.9911	2.73E-12	2.84E-11
hsa-miR-942-5p	21.72541	-1.30841	0.260941	-5.01311	5.36E-07	2.95E-06
hsa-miR-4433b-5p	150.3544	-1.31484	0.203117	-6.47325	9.59E-11	9.04E-10
hsa-miR-96-5p	21.83946	-1.31659	0.350166	-3.75884	0.000171	0.000669
hsa-let-7e-5p	377.3285	-1.31941	0.131706	-10.0178	1.27E-23	4.8E-22
hsa-miR-451a	2597.215	-1.32522	0.17326	-7.64873	2.03E-14	2.93E-13
hsa-miR-342-5p	2.021495	-1.33869	0.718251	-3.71807	0.000201	0.000763
hsa-miR-4732-3p	12.86058	-1.36548	0.39145	-3.48652	0.000489	0.001738
hsa-miR-548ap-5p/548j-5p	20.61347	-1.37234	0.271288	-5.05771	4.24E-07	2.36E-06
hsa-miR-4286	5.534063	-1.41695	0.43919	-3.22268	0.00127	0.003889
hsa-miR-451b	10.15125	-1.42581	0.4099	-3.47637	0.000508	0.001779
hsa-miR-411-5p	5.666008	-1.43274	0.507337	-2.81995	0.004803	0.012452
hsa-miR-6852-3p	6.643046	-1.45449	0.419476	-3.46383	0.000533	0.001825
hsa-miR-335-3p	3.739216	-1.48443	0.52658	-2.81304	0.004908	0.012657
hsa-miR-1294	7.697929	-1.49805	0.509521	-2.93668	0.003317	0.008981

hsa-miR-185-3p	18.01138	-1.50062	0.306935	-4.88778	1.02E-06	5.37E-06
hsa-miR-6842-3p	1.977057	-1.50347	0.604155	-2.4817	0.013076	0.03008
hsa-miR-1908-5p	7.18873	-1.52378	0.393941	-3.86606	0.000111	0.000452
hsa-miR-26b-3p	3.373266	-1.55304	0.50865	-3.04681	0.002313	0.006627
hsa-miR-29c-5p	8.660549	-1.55355	0.380551	-4.08009	4.5E-05	0.000197
hsa-miR-1976	4.203916	-1.56448	0.484142	-3.22576	0.001256	0.003872
hsa-miR-190b	10.17143	-1.56522	0.38823	-4.02902	5.6E-05	0.000243
hsa-miR-454-5p	2.848485	-1.59591	0.537304	-2.96316	0.003045	0.008336
hsa-miR-181d-5p	3.621804	-1.61189	0.573539	-2.80522	0.005028	0.012832
hsa-miR-130b-5p	2.991505	-1.63975	0.547142	-2.99086	0.002782	0.007789
hsa-miR-148b-5p	1.61401	-1.64799	0.625889	-2.62472	0.008672	0.021353
hsa-miR-1255b-5p	3.774883	-1.68579	0.598919	-2.80754	0.004992	0.012807
hsa-miR-5187-5p	4.502203	-1.71937	0.527493	-3.25518	0.001133	0.003536
hsa-miR-133a-3p	6.810987	-1.75265	0.537881	-3.25346	0.00114	0.003536
hsa-miR-151b/151a-5p	34.03798	-1.76669	0.234114	-7.54526	4.51E-14	5.82E-13
hsa-miR-628-5p	7.206595	-1.92744	0.42404	-4.54014	5.62E-06	0.000027
hsa-miR-6852-5p	3.246142	-1.99026	0.557028	-3.56345	0.000366	0.001329
hsa-miR-1275	7.914462	-2.00718	0.614106	-3.2634	0.001101	0.003458
hsa-miR-652-3p	13.57694	-2.02744	0.346976	-5.83979	5.23E-09	4.27E-08
hsa-miR-221-5p	2.419567	-2.14584	0.651715	-3.28175	0.001032	0.003261
hsa-miR-664b-5p	1.217857	-2.25105	0.811013	-2.76228	0.00574	0.014558
hsa-miR-4446-3p	1.039214	-2.40108	0.911806	-2.6265	0.008627	0.021349
hsa-miR-28-5p	1.672849	-2.44158	0.683558	-3.56109	0.000369	0.001331
hsa-miR-491-5p	3.369957	-2.48768	0.50709	-4.89557	9.8E-07	5.22E-06
hsa-miR-4433a-3p	7.041239	-2.66833	0.477484	-5.57951	2.41E-08	1.71E-07
hsa-miR-505-5p	2.826169	-2.69058	0.548359	-4.89781	9.69E-07	5.22E-06
hsa-miR-1306-3p	1.597574	-2.7015	0.773694	-3.47774	0.000506	0.001779
