# Supplementary material for: Serum microRNA Profiles and Pathways in Hepatitis B-Associated Hepatocellular Carcinoma: A South African Study
Source: Int J Mol Sci. 2024 Jan 12;25(2):975. doi: 10.3390/ijms25020975 (PMC10815595; doi:10.3390/ijms25020975)
Supplement: Supplementary file 1 [file ijms-25-00975-s001.zip › Supplementary Table S2.pdf]

**Table S2. Upregulated miRNA UMI≥ 10** (excluding miRNA in Table 1 of article)

| hsa-miRNA | UMI | LFC | Adj-P | Validated Gene Target |
|---|---|---|---|---|
| miR-3591-5p | 10.4 | 6.491 | 2.33E-16 | Upregulated in HCC |
| miR-200b-3p | 11.2 | 2.030 | 0.001 | ERG/PPP1CA/NOTCH1/ZEB1/HMGB3 |
| miR-136-3p | 12.2 | 2.617 | <0.001 | BMPR1A/B/ACVR2A/B/ |
| miR-127-5p | 14.3 | 5.291 | 8.78E-07 | TRIM25/IGF2BP3/BLVRB/SHPRH/WTAP |
| miR-122-3p | 15.1 | 6.651 | 5.55E-20 | BCL-W/SIRT1/BCL9/SLC52A2/STX6 |
| miR-1247-5p | 15.9 | 4.226 | 4.93E-07 | WNT3 |
| miR-296-3p | 16.2 | 5.480 | 2.68E-18 | MSL2/RHOA/FGRF1 |
| miR-885-5p | 17.1 | 2.806 | 1.06E-06 | CTNNB1/HK2/AEG1 |
| miR-885-3p | 17.9 | 2.750 | <0.001 | Upregulated in HBV-HCC |
| miR-665 | 20.9 | 6.247 | 1E-123 | TIMP3/ERK/MMP-9 |
| miR-9-3p | 21 | 2.829 | 1.06E-07 | HBGF-5/WWTR1/IGF1R |
| miR-323a-3p | 22.22 | 3.23 | 1.95E-05 | No direct HCC reference |
| miR-452-5p | 27.8 | 3.108 | 2.04E-12 | EPB41L3/COLEC10/TIMP3/NRP1-ABL2 |
| miR-4488 | 29.8 | 5.619 | 5.06E-21 | Cited as potential biomarker |
| miR-874-3p | 37.2 | 2.369 | 2.84E-11 | PIN1 |
| miR-210-3p | 49.7 | 2.449 | 1.06E-12 | HBx/CPEB2/SMAD4/STAT6/MMP-2/Slug/Twist1 |
| miR-494-3p | 102.4 | 3.808 | 1.07E-17 | PTEN/BMAL1/TRIM36/p27/PUMA/PTEN/TRAF3 |
| miR-34a-5p | 132.7 | 2.71 | 6.31E-37 | PEG10/TUFT1/VEGFA/C-MYC/TGF-B/YY1/PD-L1 |
| miR-4516 | 138.9 | 3.165 | 3.16E-37 | SOX5/STAT3/BCLXL |
| miR-6087 | 298.3 | 4.605 | 7.81E-57 | No direct HCC reference |

**Table S2. Downregulated miRNA ≥ 10 UMI (**excludes miRNA in Table 2 of article**)**

| hsa-miRNA | UMI | LFC | Adj-p | Validated HCC Target | Reference |
|---|---|---|---|---|---|
| miR-451b | 10.1 | -1.425 | 0.0017 | No direct HCC reference | n/a |
| miR-190b | 10.2 | -1.565 | <0.001 | IGF-1 | (64, 65) |
| miR-590-3p | 10.7 | -1.210 | 0.0064 | TEAD1/PTEN/ROCK/HMGB2/CFHR3/MDM2 | (66-71) |
| miR-4732-3p | 12.9 | -1.365 | 0.0017 | ULBP1 | (72) |
| miR-181a-3p | 12.9 | -0.961 | 0.0028 | E2F5/FAS | (73, 74) |
| miR-652-3p | 13.6 | -2.021 | 4.2E-08 | TNRC6A, biomarker | (75, 76) |
| miR-485-3p | 13.9 | -1.156 | 0.0239 | NTRK3/MAT1A | (77, 78) |
| miR-2110 | 15.3 | -0.677 | 0.0218 | No direct HCC reference | n/a |
| miR-324-3p | 16.1 | -0.783 | 0.0075 | DACT1/GLYATL1/biomarker | (79-81) |
| miR-625-5p | 16.9 | -1.294 | <0.001 | NOTCH/SNAIL1/Piezo1 | (82, 83) |
| miR-185-3p | 18.0 | -1.500 | 5.4E-06 | COL1A1 | (84) |
| miR-548j-5p | 20.6 | -1.372 | 2.4E-06 | HBx/Tg737 | (85, 86) |
| miR-942-5p | 21.7 | -1.308 | 3E-06 | ALX4/GFI1/SGTB | (87-89) |
| miR-96-5p | 21.8 | -1.316 | 0.001 | CASP9/FOXO1/IGF11/IGF1R | (90-93) |
| miR-628-3p | 22.3 | -1.217 | 9.7E-05 | Potential biomarker | (94) |

| | | | | | |
|---|---|---|---|---|---|
| miR-576-5p | 23.6 | -1.147 | <0.001 | NRIP1 | (95) |
| miR-145-5p | 23.9 | -0.597 | 0.041 | NRAS/SPATS2/KLF5/HDAC11 | (96-99) |
| miR-181a-2-3p | 26.2 | -0.616 | 0.014 | MICU1 | (100) |
| miR-1277-5p | 30.4 | -0.989 | <0.001 | No HCC reference | n/a |
| miR-151a/b-5p | 34 | -1.766 | 5.8E-13 | Potential biomarker | (101) |
| miR-140-5p | 35.3 | -1.045 | 1.1E-06 | SLUG/TGFBR1/Aurora-A/SULT2B1/FGF9/HDAC4 | (102-108) |
| miR-17-5p | 36.7 | -0.673 | 1E-05 | HSP27/INTS6/PTEN/GaINT7/C-MYC/TGFβR2/E2F1 | (109-115) |
| miR-139-3p | 53 | -1.032 | 2.4E-07 | CCL5/ANXA2R/UCK2 | (116-119) |
| miR-196b-5p | 61.1 | -1.195 | 3.1E-07 | WIPF2/NFkB/ABCB1 | (120-123) |
| miR-664a-5p | 76.8 | -0.551 | 0.002 | No direct HCC reference | n/a |
| miR-454-3p | 96 | -0.885 | 1.9E-07 | E2F/G2M/DLG5/TGF-β/FOXF2 | (124-127) |
| miR-103b | 115.6 | -1.306 | 2.8E-11 | No direct HCC reference | n/a |
| miR-409-3p | 129.1 | -0.771 | 0.001 | NF-kB/BRF2 | (128-130) |
| miR-106b-3p | 129.9 | -0.629 | <0.001 | Downregulated in HCC no gene target | (131) |
| miR-339-5p | 138.1 | -0.692 | 1.7E-07 | CDC25A/ZNF | (132-134) |
| miR-4433b-5p | 150.3 | -1.314 | 9E-10 | No direct HCC reference | n/a |
| miR-363-3p | 152.4 | -0.563 | <0.001 | SOX4/RNF38/SP1/CDK6/E2F3 | (135-139) |
| miR-625-3p | 154.9 | -0.503 | 0.002 | PLIM5/E2F1 | (140, 141) |
| miR-98-5p | 157.1 | -1.257 | 3.6E-11 | IGF2BP1/P13K/AKT/NF-kB/MAP3K2 | (142-146) |
| miR-155-5p | 161.2 | -1.128 | 3E-13 | PDK1/SOX6/PTEN/ZBTB18/CTHRC1/PD-L1 | (147-154) |
| miR-190a-5p | 187.2 | -0.821 | 5.5E-07 | HGF | (155) |
| miR-223-5p | 203.6 | -0.586 | 6.3E-05 | No direct HCC target | n/a |
| miR-744-5p | 221.4 | -1.029 | 1.8E-17 | TGF-B1 | (156) |
| miR-199b-3p | 249.7 | -0.666 | 2.2E-09 | AURKA | (157) |
| miR-182-5p | 273.3 | -0.812 | 8E-06 | FOXO3a/FOXO1/CYLD/IGF-1R/IGF-2/IGFBP-3/NPTX1 | (92, 158-162) |
| let-7e-5p | 377.3 | -1.319 | 4.8E-22 | No direct HCC reference | n/a |
| miR-584-5p | 404.9 | -0.697 | 6.9E-07 | KCNE2/CDK16/MAPK/ERK/PCK1/NRF2 | (163-166) |
| miR-15a-5p | 583.2 | -0.514 | <0.001 | GLI2/IGF1/PD1/eIF4E/BDNF/E2F3 | (167-172) |
| miR-140-3p | 710.4 | -0.53 | 1.9E-05 | GRN/PXR/HNRNPA1/VEGF-A | (173-176) |
| miR-125a-5p | 896.9 | -0.57 | 3.1E-06 | PTPN1/MAP3K11/SIRT7/VDR/MACC1/LASP1/ErbB3 | (177-183) |
| let-7d-5p | 902.5 | -0.975 | 2.9E-13 | Cited as biomarker | (184, 185) |
| let-7g-5p | 1025.9 | -0.573 | 5.6E-17 | No direct HCC reference | n/a |
| miR-425-5p | 1124.7 | -1.043 | 2.7E-21 | SCA1/PTEN/RNF11/ZC3H13/FOXD3 | (186-189) |
| miR-151a-3p | 1234.3 | -0.733 | 2.8E-10 | ATM | (190) |
| miR-451a | 2597.2 | -1.325 | 2.9E-13 | LPIN1/YWHAZ/ADAM10/HGF/MIF/MEF2D/ATF2 | (191-196) |
| miR-199a-3p | 2657.2 | -0.673 | 4.4E-11 | CD44/c-MET/YAP-1/PIK3CA/UCK2/CD151/ZHX1/PUMA/PDCD4/VEGFA/HGF/MMP2 | (197-207) |

1. Selitsky SR, Dinh TA, Toth CL, Kurtz CL, Honda M, Struck BR, et al. Transcriptomic analysis of chronic hepatitis B and C and liver cancer reveals microRNA-mediated control of cholesterol synthesis programs. MBio. 2015;6(6):e01500-15.
2. Moh-Moh-Aung A, Fujisawa M, Ito S, Katayama H, Ohara T, Ota Y, et al. Decreased miR-200b-3p in cancer cells leads to angiogenesis in HCC by enhancing endothelial ERG expression. Scientific reports. 2020;10(1):10418.
3. Sekar D. H19 Promotes HCC Bone Metastasis by Reducing Osteoprotegerin Expression in a PPP1CA/p38MAPK‐Dependent Manner and Sponging miR‐200b‐3p. Hepatology. 2021;74(3):1713.
4. Qiu W, Wang Z, Chen R, Shi H, Ma Y, Zhou H, et al. Xiaoai Jiedu Recipe suppresses hepatocellular carcinogenesis through the miR‐200b-3p/Notch1 axis. Cancer Management and Research. 2020:11121-31.
5. Xu Y, Luan G, Liu F, Zhang Y, Li Z, Liu Z, et al. Exosomal miR-200b-3p induce macrophage polarization by regulating transcriptional repressor ZEB1 in hepatocellular carcinoma. Hepatology International. 2023:1-15.
6. Wang L-k, Xie X-N, Song X-H, Su T, Chang X-L, Xu M, et al. Upregulation of miR-200b inhibits hepatocellular carcinoma cell proliferation and migration by targeting HMGB3 protein. Technology in cancer research & treatment. 2018;17:1533033818806475.
7. Majumdar S, Chakraborty A, Das S, Gorain M, Chatterjee S, Bhowmik S, et al. Sponging of Five Convergent Tumour Suppressor MicroRNAs by lncRNA-KCNQ1OT1 Promotes Hepatocellular Carcinoma. Journal of Clinical and Experimental Hepatology. 2023;13:S1.
8. Banerjee S, Majumdar S, Chakraborty A, Das S, Gorain M, Chatterjee S, et al. Sponging of five tumour suppressor miRNAs by lncRNA-KCNQ1OT1 activates BMPR1A/BMPR1B-ACVR2A/ACVR2B signalling and promotes chemoresistance to hepatocellular carcinoma. 2023.
9. Zhang W, Zhu L, Yang G, Zhou B, Wang J, Qu X, et al. Hsa_circ_0026134 expression promoted TRIM25-and IGF2BP3-mediated hepatocellular carcinoma cell proliferation and invasion via sponging miR-127-5p. Bioscience reports. 2020;40(7).
10. Huan L, Bao C, Chen D, Li Y, Lian J, Ding J, et al. MicroRNA‐127‐5p targets the biliverdin reductase B/nuclear factor‐κB pathway to suppress cell growth in hepatocellular carcinoma cells. Cancer Science. 2016;107(3):258-66.
11. Su Y, Xu C, Liu Y, Hu Y, Wu H. Circular RNA hsa_circ_0001649 inhibits hepatocellular carcinoma progression via multiple miRNAs sponge. Aging (Albany NY). 2019;11(10):3362.
12. Hu Z, Chen G, Zhao Y, Gao H, Li L, Yin Y, et al. Exosome-derived circCCAR1 promotes CD8+ T-cell dysfunction and anti-PD1 resistance in hepatocellular carcinoma. Molecular Cancer. 2023;22(1):1-21.
13. Qin L, Huang J, Wang G, Huang J, Wu X, Li J, et al. Integrated analysis of clinical significance and functional involvement of microRNAs in hepatocellular carcinoma. Journal of cellular physiology. 2019;234(12):23581-95.
14. Liang H-W, Wang N, Wang Y, Wang F, Fu Z, Yan X, et al. Hepatitis B virus-human chimeric transcript HBx-LINE1 promotes hepatic injury via sequestering cellular microRNA-122. Journal of hepatology. 2016;64(2):278-91.
15. Budhu A, Jia HL, Forgues M, Liu CG, Goldstein D, Lam A, et al. Identification of metastasis‐related microRNAs in hepatocellular carcinoma. Hepatology. 2008;47(3):897-907.
16. Lin CJ-F, Gong H-Y, Tseng H-C, Wang W-L, Wu J-L. miR-122 targets an anti-apoptotic gene, Bcl-w, in human hepatocellular carcinoma cell lines. Biochemical and biophysical research communications. 2008;375(3):315-20.
17. Mjelle R, Dima SO, Bacalbasa N, Chawla K, Sorop A, Cucu D, et al. Comprehensive transcriptomic analyses of tissue, serum, and serum exosomes from hepatocellular carcinoma patients. BMC cancer. 2019;19:1-13.

18. Shi Y, Huang A. Effects of sorafenib on lung metastasis in rats with hepatocellular carcinoma: the role of microRNAs. Tumor Biology. 2015;36:8455-63.
19. Xu J, Zhu X, Wu L, Yang R, Yang Z, Wang Q, et al. MicroRNA‐122 suppresses cell proliferation and induces cell apoptosis in hepatocellular carcinoma by directly targeting Wnt/β‐catenin pathway. Liver international. 2012;32(5):752-60.
20. Xu J, Wu C, Che X, Wang L, Yu D, Zhang T, et al. Circulating MicroRNAs, miR‐21, miR‐122, and miR‐223, in patients with hepatocellular carcinoma or chronic hepatitis. Molecular carcinogenesis. 2011;50(2):136-42.
21. López-Sánchez GN, Montalvo-Javé E, Domínguez-Perez M, Antuna-Puente B, Beltrán-Anaya FO, Hidalgo-Miranda A, et al. Hepatic mir-122-3p, mir-140-5p and mir-148b-5p expressions are correlated with cytokeratin-18 serum levels in MAFLD. Annals of Hepatology. 2022;27(6):100756.
22. Luna JM, Barajas JM, Teng K-y, Sun H-L, Moore MJ, Rice CM, et al. Argonaute CLIP defines a deregulated miR-122-bound transcriptome that correlates with patient survival in human liver cancer. Molecular cell. 2017;67(3):400-10. e7.
23. Cui Y-H, Li H-Y, Gao Z-X, Liang N, Ma S-S, Meng F-J, et al. Regulation of apoptosis by miR-122 in pterygium via targeting Bcl-w. Investigative ophthalmology & visual science. 2016;57(8):3723-30.
24. Chu Y, Fan W, Guo W, Zhang Y, Wang L, Guo L, et al. miR-1247-5p functions as a tumor suppressor in human hepatocellular carcinoma by targeting Wnt3. Oncology reports. 2017;38(1):343-51.
25. Li X, An M, Gao Z. In hepatocellular carcinoma, miRNA-296-3p targets MSL2 and suppresses cell proliferation and invasion. Journal of Oncology. 2021;2021.
26. Yu L-X, Zhang B-L, Yang Y, Wang M-C, Lei G-L, Gao Y, et al. Exosomal microRNAs as potential biomarkers for cancer cell migration and prognosis in hepatocellular carcinoma patient-derived cell models. Oncology reports. 2019;41(1):257-69.
27. Wang L, Bo X, Zheng Q, Xiao X, Wu L, Li B. miR‐296 inhibits proliferation and induces apoptosis by targeting FGFR 1 in human hepatocellular carcinoma. FEBS letters. 2016;590(23):4252-62.
28. Zhang Z, Yin J, Yang J, Shen W, Zhang C, Mou W, et al. miR-885-5p suppresses hepatocellular carcinoma metastasis and inhibits Wnt/β-catenin signaling pathway. Oncotarget. 2016;7(46):75038.
29. Xu F, Yan J-J, Gan Y, Chang Y, Wang H-L, He X-X, et al. miR-885-5p negatively regulates warburg effect by silencing hexokinase 2 in liver cancer. Molecular Therapy-Nucleic Acids. 2019;18:308-19.
30. Li C, Wang X, Song Q. MicroRNA 885-5p inhibits hepatocellular carcinoma metastasis by repressing AEG1. OncoTargets and therapy. 2020:981-8.
31. Lee SJ, Ko EJ, Yeon JE, Yoo YJ, Kang KH, Yoon EL, et al. Plenary Session lI: MicroRNA Profile in Chronic Hepatitis B-related Hepatocellular Carcinoma (HCC): Overexpression of miR-224 in HCC Tissue Predicts Recurrence after Curative Resection. 춘· 추계 학술대회 (KASL). 2013;2013(1):11-2.
32. Liu H, Yan Y, Lin J, He C, Liao H, Li H, et al. Circular RNA circSFMBT2 downregulation by HBx promotes hepatocellular carcinoma metastasis via the miR-665/TIMP3 axis. Molecular Therapy-Nucleic Acids. 2022;29:788-802.
33. Qu Z, Wu J, Wu J, Ji A, Qiang G, Jiang Y, et al. Exosomal miR-665 as a novel minimally invasive biomarker for hepatocellular carcinoma diagnosis and prognosis. Oncotarget. 2017;8(46):80666.
34. Zhu X, Peng C, Peng Z, Chang R, Guo Q. Sevoflurane inhibits metastasis in hepatocellular carcinoma by inhibiting MiR-665-induced activation of the ERK/MMP pathway. Cell Transplantation. 2022;31:09636897221104447.
35. Tang J, Li Y, Liu K, Zhu Q, Yang W-H, Xiong L-K, et al. Exosomal miR-9-3p suppresses HBGF-5 expression and is a functional biomarker in hepatocellular carcinoma. Minerva medica. 2017;109(1):15-23.

36. Higashi T, Hayashi H, Ishimoto T, Takeyama H, Kaida T, Arima K, et al. miR-9-3p plays a tumour-suppressor role by targeting TAZ (WWTR1) in hepatocellular carcinoma cells. British journal of cancer. 2015;113(2):252-8.
37. Guan Q, Yuan B, Zhang X, Yan T, Li J, Xu W. Long non-coding RNA DUXAP8 promotes tumorigenesis by regulating IGF1R via miR-9-3p in hepatocellular carcinoma. Experimental and Therapeutic Medicine. 2021;22(1):1-12.
38. Caviglia G, Fagoonee S. Role and function of exosomal miR-9-3p in hepatocellular carcinoma. Minerva Medica. 2018;109(1):4-6.
39. Zhu L, Yang N, Chen J, Zeng T, Yan S, Liu Y, et al. LINC00052 upregulates EPB41L3 to inhibit migration and invasion of hepatocellular carcinoma by binding miR-452-5p. Oncotarget. 2017;8(38):63724.
40. Zheng J, Cheng D, Wu D, Wang L, Qu F, Wu X, et al. MiR-452-5p mediates the proliferation, migration and invasion of hepatocellular carcinoma cells via targeting COLEC10. Personalized Medicine. 2021;18(2):97-106.
41. Zongqiang H, Jiapeng C, Yingpeng Z, Chuntao Y, Yiting W, Jiashun Z, et al. Exosomal miR-452-5p induce M2 macrophage polarization to accelerate hepatocellular carcinoma progression by targeting TIMP3. Journal of Immunology Research. 2022;2022.
42. Rong M-H, Cai K-T, Lu H-P, Guo Y-N, Huang X-Y, Zhu Z-H, et al. Overexpression of MiR-452-5p in hepatocellular carcinoma tissues and its prospective signaling pathways. International Journal of Clinical and Experimental Pathology. 2019;12(11):4041.
43. Yang W, Ju H-Y, Tian X-F. Circular RNA-ABCB10 suppresses hepatocellular carcinoma progression through upregulating NRP1/ABL2 via sponging miR-340-5p/miR-452-5p. European Review for Medical & Pharmacological Sciences. 2020;24(5).
44. El-Shqnqery HE, Mohamed RH, Samir O, Ayoub I, El-Sayed WM, Sayed AA. miRNome of Child A hepatocellular carcinoma in Egyptian patients. Frontiers in Oncology. 2023;13.
45. Leong K-W, Cheng C-W, Wong C-M, Oi-Lin IN, Kwong Y-L, Tse E. miR-874-3p is down-regulated in hepatocellular carcinoma and negatively regulates PIN1 expression. Oncotarget. 2017;8(7):11343.
46. Morishita A, Fujita K, Iwama H, Chiyo T, Fujihara S, Oura K, et al. Role of microRNA-210-3p in hepatitis B virus-related hepatocellular carcinoma. American Journal of Physiology-Gastrointestinal and Liver Physiology. 2020;318(3):G401-G9.
47. You R, Yang Y, Yin G, Jiang H, Lu Y, Gui L, et al. CPEB2 Suppresses Hepatocellular Carcinoma Epithelial–Mesenchymal Transition and Metastasis through Regulating the HIF-1$\alpha$/miR-210-3p/CPEB2 Axis. Pharmaceutics. 2023;15(7):1887.
48. Lin X-J, Fang J-H, Yang X-J, Zhang C, Yuan Y, Zheng L, et al. Hepatocellular carcinoma cell-secreted exosomal microRNA-210 promotes angiogenesis in vitro and in vivo. Molecular Therapy-Nucleic Acids. 2018;11:243-52.
49. Li X, Wu H, Wu M, Feng Y, Wu S, Shen X, et al. Hypoxia-related miR-210-5p and miR-210-3p regulate hypoxia-induced migration and epithelial-mesenchymal transition in hepatoma cells. Int J Clin Exp Med. 2019;12:5096-104.
50. Lin H, Huang Z-P, Liu J, Qiu Y, Tao Y-p, Wang M-c, et al. MiR-494-3p promotes PI3K/AKT pathway hyperactivation and human hepatocellular carcinoma progression by targeting PTEN. Scientific reports. 2018;8(1):10461.
51. Yang Y, Yang T, Zhao Z, Zhang H, Yuan P, Wang G, et al. Down-regulation of BMAL1 by MiR-494-3p Promotes Hepatocellular Carcinoma Growth and Metastasis by Increasing GPAM-mediated Lipid Biosynthesis. International Journal of Biological Sciences. 2022;18(16):6129.
52. Yan H, Ma X, Mi Z, He Z, Rong P. Extracellular polysaccharide from Rhizopus nigricans inhibits hepatocellular carcinoma via miR-494-3p/TRIM36 axis and cyclin E ubiquitination. Journal of Clinical and Translational Hepatology. 2022;10(4):608.

53. Pollutri D, Patrizi C, Marinelli S, Giovannini C, Trombetta E, Giannone FA, et al. The epigenetically regulated miR-494 associates with stem-cell phenotype and induces sorafenib resistance in hepatocellular carcinoma. Cell death & disease. 2018;9(1):4.
54. Li H, Zhang L, Cai N, Zhang B, Sun S. MicroRNA-494-3p prevents liver fibrosis and attenuates hepatic stellate cell activation by inhibiting proliferation and inducing apoptosis through targeting TRAF3. Annals of Hepatology. 2021;23:100305.
55. Li Y, Guo D, Lu G, Mohiuddin Chowdhury ATM, Zhang D, Ren M, et al. LncRNA SNAI3-AS1 promotes PEG10-mediated proliferation and metastasis via decoying of miR-27a-3p and miR-34a-5p in hepatocellular carcinoma. Cell Death & Disease. 2020;11(8):685.
56. Niu X, Wei N, Peng L, Li X, Zhang X, Wang C. miR-34a-5p plays an inhibitory role in hepatocellular carcinoma by regulating target gene VEGFA. Malays J Pathol. 2022;44(1):39-52.
57. Zhao X, Zhuang Y, Wang B, Yuan B, Du S, Zeng Z. The miR-34a-5p-c-MYC-CHK1/CHK2 Axis Counteracts Cancer Stem Cell-Like Properties and Enhances Radiosensitivity in Hepatocellular Cancer Through Repression of the DNA Damage Response. Radiat Res. 2023;199(1):48-60.
58. Feili X, Wu S, Ye W, Tu J, Lou L. MicroRNA-34a-5p inhibits liver fibrosis by regulating TGF-β1/Smad3 pathway in hepatic stellate cells. Cell Biol Int. 2018;42(10):1370-6.
59. Xiao Z, Liu Y, Zhao J, Li L, Hu L, Lu Q, et al. Long noncoding RNA LINC01123 promotes the proliferation and invasion of hepatocellular carcinoma cells by modulating the miR-34a-5p/TUFT1 axis. International Journal of Biological Sciences. 2020;16(13):2296.
60. Xu X-P, Peng X-Q, Yin X-M, Liu Y, Shi Z-Y. miR-34a-5p suppresses the invasion and metastasis of liver cancer by targeting the transcription factor YY1 to mediate MYCT1 upregulation. Acta Histochemica. 2020;122(6):151576.
61. He T, Huang J, Liang H, Zhong B, Xu G, Zhu X. Circular RNA hsa_circ_0005239 contributes to hepatocellular carcinoma cell migration, invasion, and angiogenesis by targeting the miR-34a-5p/PD-L1 axis. Cell Biol Int. 2023.
62. Li Q, Wang W, Yang T, Li D, Huang Y, Bai G, et al. LINC00520 up-regulates SOX5 to promote cell proliferation and invasion by miR-4516 in human hepatocellular carcinoma. Biological Chemistry. 2022;403(7):665-78.
63. Li O, Li Z, Tang Q, Li Y, Yuan S, Shen Y, et al. Long stress induced non-coding transcripts 5 (LSINCT5) promotes hepatocellular carcinoma progression through interaction with high-mobility group AT-hook 2 and MiR-4516. Medical Science Monitor: International Medical Journal of Experimental and Clinical Research. 2018;24:8510.
64. Hung T-M, Ho C-M, Liu Y-C, Lee J-L, Liao Y-R, Wu Y-M, et al. Up-regulation of microRNA-190b plays a role for decreased IGF-1 that induces insulin resistance in human hepatocellular carcinoma. PloS one. 2014;9(2):e89446.
65. Xie Y, Wang Y, Xue W, Zou H, Li K, Liu K, et al. Profiling and integrated analysis of differentially expressed microRNAs as novel biomarkers of hepatocellular carcinoma. Frontiers in Oncology. 2022;11:770918.
66. Ge X, Gong L. MiR-590-3p suppresses hepatocellular carcinoma growth by targeting TEAD1. Tumor Biology. 2017;39(3):1010428317695947.
67. Yang H, Zheng W, Zhao W, Guan C, An J. Roles of miR-590-5p and miR-590-3p in the development of hepatocellular carcinoma. Nan fang yi ke da xue xue bao= Journal of Southern Medical University. 2013;33(6):804-11.
68. You L-N, Tai Q-W, Xu L, Hao Y, Guo W-J, Zhang Q, et al. Exosomal LINC00161 promotes angiogenesis and metastasis via regulating miR-590-3p/ROCK axis in hepatocellular carcinoma. Cancer Gene Therapy. 2021;28(6):719-36.

69. Pu J, Tan C, Shao Z, Wu X, Zhang Y, Xu Z, et al. Long noncoding RNA PART1 promotes hepatocellular carcinoma progression via targeting miR-590-3p/HMGB2 axis. OncoTargets and therapy. 2020:9203-11.
70. Wan Z, Li X, Luo X, Wang B, Zhou X, Chen A. The miR-590-3p/CFHR3/STAT3 signaling pathway promotes cell proliferation and metastasis in hepatocellular carcinoma. Aging (Albany NY). 2022;14(14):5783.
71. Youssef AI, Khaled GM, Amleh A. Functional role and epithelial to mesenchymal transition of the miR-590-3p/MDM2 axis in hepatocellular carcinoma. BMC cancer. 2023;23(1):396.
72. Qi F, Du X, Zhao Z, Zhang D, Huang M, Bai Y, et al. Tumor mutation burden-associated LINC00638/miR-4732-3p/ULBP1 axis promotes immune escape via PD-L1 in hepatocellular carcinoma. Frontiers in Oncology. 2021;11:729340.
73. Zou C, Chen J, Chen K, Wang S, Cao Y, Zhang J, et al. Functional analysis of miR-181a and Fas involved in hepatitis B virus-related hepatocellular carcinoma pathogenesis. Experimental cell research. 2015;331(2):352-61.
74. Zou C, Li Y, Cao Y, Zhang J, Jiang J, Sheng Y, et al. Up-regulated MicroRNA-181a induces carcinogenesis in Hepatitis B virus-related hepatocellular carcinoma by targeting E2F5. BMC cancer. 2014;14(1):97.
75. Chen W, Ru J, Wu T, Man D, Wu J, Wu L, et al. MiR-652-3p promotes malignancy and metastasis of cancer cells via inhibiting TNRC6A in hepatocellular carcinoma. Biochemical and Biophysical Research Communications. 2023;640:1-11.
76. Chi X, Jiang Y, Chen Y, Lv L, Chen J, Yang F, et al. Upregulation of microRNA miR-652-3p is a prognostic risk factor for hepatocellular carcinoma and regulates cell proliferation, migration, and invasion. Bioengineered. 2021;12(1):7519-28.
77. Xiong D, Sheng Y, Ding S, Chen J, Tan X, Zeng T, et al. LINC00052 regulates the expression of NTRK3 by miR-128 and miR-485-3p to strengthen HCC cells invasion and migration. Oncotarget. 2016;7(30):47593.
78. Yang H, Cho ME, Li TW, Peng H, Ko KS, Mato JM, et al. MicroRNAs regulate methionine adenosyltransferase 1A expression in hepatocellular carcinoma. The Journal of clinical investigation. 2012;123(1).
79. Tuo H, Wang Y, Wang L, Yao B, Li Q, Wang C, et al. MiR-324-3p promotes tumor growth through targeting DACT1 and activation of Wnt/β-catenin pathway in hepatocellular carcinoma. Oncotarget. 2017;8(39):65687.
80. Bao L, Li P, Zhao H, Chen L, Wang Y, Liang S, et al. Pseudogene PLGLA exerts anti-tumor effects on hepatocellular carcinoma through modulating miR-324–3p/GLYATL1 axis. Digestive and Liver Disease. 2022;54(7):918-26.
81. Zhao L, Yang Q, Liu J, editors. Clinical Value Evaluation of microRNA-324-3p and Other Available Biomarkers in Patients With HBV Infection–Related Hepatocellular Carcinoma. Open Forum Infectious Diseases; 2021: Oxford University Press US.
82. Mo Z, Li R, Cao C, Li Y, Zheng S, Wu R, et al. Splicing factor SNRPA associated with microvascular invasion promotes hepatocellular carcinoma metastasis through activating NOTCH1/Snail pathway and is mediated by circSEC62/miR‐625‐5p axis. Environmental Toxicology. 2023;38(5):1022-37.
83. Li M, Zhang X, Wang M, Wang Y, Qian J, Xing X, et al. Activation of Piezo1 contributes to matrix stiffness‐induced angiogenesis in hepatocellular carcinoma. Cancer Communications. 2022;42(11):1162-84.
84. Li B, Zhou J, Luo Y, Tao K, Zhang L, Zhao Y, et al. Suppressing circ_0008494 inhibits HSCs activation by regulating the miR-185-3p/Col1a1 axis. Frontiers in Pharmacology. 2022;13:1050093.

85. Hu XM, Yan XH, Hu YW, Huang JL, Cao SW, Ren TY, et al. miRNA‐548p suppresses hepatitis B virus X protein associated hepatocellular carcinoma by downregulating oncoprotein hepatitis B x‐interacting protein. Hepatology Research. 2016;46(8):804-15.
86. Zhao G, Wang T, Huang Q-K, Pu M, Sun W, Zhang Z-C, et al. MicroRNA-548a-5p promotes proliferation and inhibits apoptosis in hepatocellular carcinoma cells by targeting Tg737. World journal of gastroenterology. 2016;22(23):5364.
87. Xu Q, Zhou L, Yang G, Meng F, Wan Y, Wang L, et al. Overexpression of circ_0001445 decelerates hepatocellular carcinoma progression by regulating miR-942-5p/ALX4 axis. Biotechnology Letters. 2020;42:2735-47.
88. Lu L, Li S, Zhang Y, Luo Z, Chen Y, Ma J, et al. GFI1-mediated upregulation of LINC00675 as a ceRNA restrains hepatocellular carcinoma metastasis by sponging miR-942-5p. Frontiers in Oncology. 2021;10:607593.
89. Yang S, Wang D, Zhang R. Circ_0102543 suppresses hepatocellular carcinoma progression through the miR‐942‐5p/SGTB axis. Annals of Gastroenterological Surgery. 2023.
90. Iwai N, Yasui K, Tomie A, Gen Y, Terasaki K, Kitaichi T, et al. Oncogenic miR-96-5p inhibits apoptosis by targeting the caspase-9 gene in hepatocellular carcinoma. International journal of oncology. 2018;53(1):237-45.
91. Yuan F, Tang Y, Cao M, Ren Y, Li Y, Yang G, et al. Identification of the hsa_circ_0039466/miR-96-5p/FOXO1 regulatory network in hepatocellular carcinoma by whole-transcriptome analysis. Annals of Translational Medicine. 2022;10(14).
92. Assal RA, El Tayebi HM, Hosny KA, Esmat G, Abdelaziz AI. A pleiotropic effect of the single clustered hepatic metastamiRs miR-96-5p and miR-182-5p on insulin-like growth factor II, insulin-like growth factor-1 receptor and insulin-like growth factor-binding protein-3 in hepatocellular carcinoma. Molecular medicine reports. 2015;12(1):645-50.
93. Matsui T, Hamada‐Tsutsumi S, Naito Y, Nojima M, Iio E, Tamori A, et al. Identification of microRNA‐96‐5p as a postoperative, prognostic microRNA predictor in nonviral hepatocellular carcinoma. Hepatology Research. 2022;52(1):93-104.
94. Sheng L, Li J, Qin H, Liu L, Zhang D, Zhang Q, et al. Blood exosomal miRNA profiling reveals the complexity of hepatocellular carcinoma and identifies potential biomarkers as differential diagnosis. 2020.
95. Cui Y, Lin H, Zhao Y, Liu J, Zhang C, Wen F, et al. A Novel Long Non-coding RNA, ENSG00000236199, Inhibits Proliferation and Metastasis through NRIP1 by Sponging miR-576-5p in Hepatocellular Carcinoma. 2020.
96. Ding B, Fan W, Lou W. hsa_circ_0001955 enhances in vitro proliferation, migration, and invasion of HCC cells through miR-145-5p/NRAS axis. Molecular Therapy-Nucleic Acids. 2020;22:445-55.
97. Dong G, Zhang S, Shen S, Sun L, Wang X, Wang H, et al. SPATS2, negatively regulated by miR-145-5p, promotes hepatocellular carcinoma progression through regulating cell cycle. Cell Death & Disease. 2020;11(10):837.
98. Liang H, Sun H, Yang J, Yi C. miR-145-5p reduces proliferation and migration of hepatocellular carcinoma by targeting KLF5. Molecular Medicine Reports. 2018;17(6):8332-8.
99. Wang W, Ding B, Lou W, Lin S. Promoter hypomethylation and miR-145-5p downregulation-mediated HDAC11 overexpression promotes sorafenib resistance and metastasis of hepatocellular carcinoma cells. Frontiers in cell and developmental biology. 2020;8:724.
100. Dong Z, Yang X, Qiu T, Zhang G, Li Q, Jiang L, et al. Exosomal miR-181a-2-3p derived from citreoviridin-treated hepatocytes activates hepatic stellate cells trough inducing mitochondrial calcium overload. Chemico-Biological Interactions. 2022;358:109899.

101. Weis A, Marquart L, Calvopina DA, Genz B, Ramm GA, Skoien R. Serum microRNAs as biomarkers in hepatitis C: preliminary evidence of a microRNA panel for the diagnosis of hepatocellular carcinoma. International journal of molecular sciences. 2019;20(4):864.
102. Lv J, Fan H-x, Zhao X-p, Lv P, Fan J-y, Zhang Y, et al. Long non-coding RNA Unigene56159 promotes epithelial–mesenchymal transition by acting as a ceRNA of miR-140-5p in hepatocellular carcinoma cells. Cancer letters. 2016;382(2):166-75.
103. Li Y, Liu G, Li X, Dong H, Xiao W, Lu S. Long non-coding RNA SBF2-AS1 promotes hepatocellular carcinoma progression through regulation of miR-140-5p-TGFBR1 pathway. Biochemical and biophysical research communications. 2018;503(4):2826-32.
104. Fan L, Huang X, Chen J, Zhang K, Gu Y-h, Sun J, et al. Long noncoding RNA MALAT1 contributes to sorafenib resistance by targeting miR-140-5p/Aurora-A signaling in hepatocellular carcinoma. Molecular cancer therapeutics. 2020;19(5):1197-209.
105. Wang ZY, Zhu Z, Wang HF, Qin B, Liu J, Yao XH, et al. Downregulation of circDYNC1H1 exhibits inhibitor effect on cell proliferation and migration in hepatocellular carcinoma through miR‐140‐5p. Journal of Cellular Physiology. 2019;234(10):17775-85.
106. Binswanger H. Empowering rural people for their own development. Farewell lecture from the World Bank; 2004.
107. Yang H, Fang F, Chang R, Yang L. MicroRNA‐140‐5p suppresses tumor growth and metastasis by targeting transforming growth factor β receptor 1 and fibroblast growth factor 9 in hepatocellular carcinoma. Hepatology. 2013;58(1):205-17.
108. Wu L, Li H, Chen S, Wu X, Chen X, Wang F. Catalpol inhibits the proliferation, migration and metastasis of HCC cells by regulating miR-140-5p expression. Molecular medicine reports. 2021;23(1):1-.
109. Chen L, Jiang M, Yuan W, Tang H. miR-17-5p as a novel prognostic marker for hepatocellular carcinoma. Journal of Investigative Surgery. 2012;25(3):156-61.
110. Yang F, Yin Y, Wang F, Wang Y, Zhang L, Tang Y, et al. miR‐17‐5p Promotes migration of human hepatocellular carcinoma cells through the p38 mitogen‐activated protein kinase‐heat shock protein 27 pathway. Hepatology. 2010;51(5):1614-23.
111. Peng H, Ishida M, Li L, Saito A, Kamiya A, Hamilton JP, et al. Pseudogene INTS6P1 regulates its cognate gene INTS6 through competitive binding of miR-17-5p in hepatocellular carcinoma. Oncotarget. 2015;6(8):5666.
112. Shan SW, Fang L, Shatseva T, Rutnam ZJ, Yang X, Du W, et al. Mature miR-17-5p and passenger miR-17-3p induce hepatocellular carcinoma by targeting PTEN, GalNT7 and vimentin in different signal pathways. Journal of cell science. 2013;126(6):1517-30.
113. Liu D, Dong L, Liu Y, Wen D, Gao D, Sun H, et al. A c-Myc/miR-17-5p feedback loop regulates metastasis and invasion of hepatocellular carcinoma. Tumor Biology. 2016;37:5039-47.
114. Liu H-T, Luo C-P, Jiang M-J, Deng Z-J, Teng Y-X, Su J-Y, et al. miR-17-5p slows progression of hepatocellular carcinoma by downregulating TGFβR2. Clinical and Translational Oncology. 2023:1-12.
115. El Tayebi H, Omar K, Hegy S, El Maghrabi M, El Brolosy M, Hosny K, et al. Repression of miR-17-5p with elevated expression of E2F-1 and c-MYC in non-metastatic hepatocellular carcinoma and enhancement of cell growth upon reversing this expression pattern. Biochemical and biophysical research communications. 2013;434(3):421-7.
116. Cao P, Ma B, Sun D, Zhang W, Qiu J, Qin L, et al. hsa_circ_0003410 promotes hepatocellular carcinoma progression by increasing the ratio of M2/M1 macrophages through the miR‐139‐3p/CCL5 axis. Cancer Science. 2022;113(2):634-47.
117. Zhu Y, Zhou C, He Q. High miR-139-3p expression predicts a better prognosis for hepatocellular carcinoma: a pooled analysis. Journal of International Medical Research. 2019;47(1):383-90.

118. Zou ZC, Dai M, Huang ZY, Lu Y, Xie HP, Li YF, et al. MicroRNA-139-3p suppresses tumor growth and metastasis in hepatocellular carcinoma by repressing ANXA2R. Oncology research. 2018;26(9):1391.
119. Yu Y, You S, Fan R, Shan X. UCK2 regulated by miR-139-3p regulates the progression of hepatocellular carcinoma cells. Future Oncology. 2022;18(8):979-90.
120. Zhai H, Zhang X, Chen S, Fan M, Ma S, Sun X. RP5-1120P11. 3 promotes hepatocellular carcinoma development via the miR-196b-5p–WIPF2 axis. Biochemistry and Cell Biology. 2020;98(2):238-48.
121. Zhang L, Luo B, Dang Y-w, He R-q, Peng Z-g, Chen G, et al. Clinical significance of microRNA-196b-5p in hepatocellular carcinoma and its potential molecular mechanism. Journal of Cancer. 2019;10(22):5355.
122. Ando Y, Yang G-X, Kenny TP, Kawata K, Zhang W, Huang W, et al. Overexpression of microRNA-21 is associated with elevated pro-inflammatory cytokines in dominant-negative TGF-β receptor type II mouse. Journal of autoimmunity. 2013;41:111-9.
123. Pu B, Cao Y, Li Y, Tang L, Xia J, Li B. MiR-196b-5p regulates the proliferation of drug-resistant hepatocellular carcinoma cell lines by activating NFκB/ABCB1 signaling pathway. Tropical Journal of Pharmaceutical Research. 2020;19(1):39-44.
124. Li Y, Jiao Y, Fu Z, Luo Z, Su J, Li Y. High miR-454-3p expression predicts poor prognosis in hepatocellular carcinoma. Cancer management and research. 2019:2795-802.
125. Dong Y, Wang Q, Sun J, Liu H, Wang H. Long non-coding RNA TPTEP1 exerts inhibitory effects on hepatocellular carcinoma by impairing microRNA-454-3p-mediated DLG5 downregulation. Digestive and Liver Disease. 2022;54(2):268-79.
126. Liu H, Zheng J, Yang S, Zong Q, Wang Z, Liao X. Mir-454-3p induced WTX deficiency promotes hepatocellular carcinoma progressions through regulating TGF-β signaling pathway. Journal of Cancer. 2022;13(6):1820.
127. Shi D, Li H, Zhang J, Li Y. CircGDI2 regulates the proliferation, migration, invasion and apoptosis of OSCC via miR-454-3p/FOXF2 axis. Cancer Management and Research. 2021:1371-82.
128. Li L, Ai R, Yuan X, Dong S, Zhao D, Sun X, et al. LINC00886 Facilitates Hepatocellular Carcinoma Tumorigenesis by Sequestering microRNA-409-3p and microRNA-214-5p. Journal of Hepatocellular Carcinoma. 2023:863-81.
129. Chang J-H, Xu B-W, Shen D, Zhao W, Wang Y, Liu J-l, et al. BRF2 is mediated by microRNA-409-3p and promotes invasion and metastasis of HCC through the Wnt/β-catenin pathway. Cancer Cell International. 2023;23(1):1-12.
130. Long Z, Gong F, Li Y, Fan Z, Li J. Circ_0000285 regulates proliferation, migration, invasion and apoptosis of osteosarcoma by miR-409-3p/IGFBP3 axis. Cancer cell international. 2020;20:1-11.
131. Moshiri F, Salvi A, Gramantieri L, Sangiovanni A, Guerriero P, De Petro G, et al. Circulating miR-106b-3p, miR-101-3p and miR-1246 as diagnostic biomarkers of hepatocellular carcinoma. Oncotarget. 2018;9(20):15350.
132. Wang Y-L, Chen C-m, Wang X-M, Wang L. Effects of miR-339-5p on invasion and prognosis of hepatocellular carcinoma. Clinics and research in hepatology and gastroenterology. 2016;40(1):51-6.
133. Zeng H, Zheng J, Wen S, Luo J, Shao G, Zhang Y. MicroRNA-339 inhibits human hepatocellular carcinoma proliferation and invasion via targeting ZNF689. Drug design, development and therapy. 2019:435-45.
134. Shi Y, Guo J, Ye A, Zhou J, Zheng L, Cai J. Knockdown of Long Non-Coding RNA (LncRNA) MAFG Divergent Transcript Inhibits the Tumorigenesis of Hepatocellular Carcinoma Through miR-339-5p/CDC25A Axis. Journal of Biomedical Nanotechnology. 2023;19(2):195-205.

135. Wang J, Tang Q, Lu L, Luo Z, Li W, Lu Y, et al. LncRNA OIP5-AS1 interacts with miR-363-3p to contribute to hepatocellular carcinoma progression through up-regulation of SOX4. Gene Therapy. 2020;27(10-11):495-504.
136. Hu P-A, Miao Y-Y, Yu S, Guo N. Long non-coding RNA SNHG5 promotes human hepatocellular carcinoma progression by regulating miR-363-3p/RNF38 axis. European Review for Medical & Pharmacological Sciences. 2020;24(7).
137. Ying J, Yu X, Ma C, Zhang Y, Dong J. MicroRNA-363-3p is downregulated in hepatocellular carcinoma and inhibits tumorigenesis by directly targeting specificity protein 1. Molecular medicine reports. 2017;16(2):1603-11.
138. Lu Y-B, Jiang Q, Yang M-Y, Zhou J-X, Zhang Q. Long noncoding RNA NNT-AS1 promotes hepatocellular carcinoma progression and metastasis through miR-363/CDK6 axis. Oncotarget. 2017;8(51):88804.
139. Ye J, Zhang W, Liu S, Liu Y, Liu K. miR-363 inhibits the growth, migration and invasion of hepatocellular carcinoma cells by regulating E2F3 Retraction in/10.3892/or. 2021.8181. Oncology reports. 2017;38(6):3677-84.
140. Wang J, Zhu Y, Ai X, Wan H, Jia W, Chu J, et al. Long noncoding RNA 02027 inhibits proliferation, migration and invasion of hepatocellular carcinoma via miR‐625‐3p/PDLIM5 pathway. The Journal of Gene Medicine. 2023:e3485.
141. Chen S. LINC00852 regulates cell proliferation, invasion, migration and apoptosis in hepatocellular carcinoma Via the miR-625/E2F1 Axis. Cellular and Molecular Bioengineering. 2022;15(2):207-17.
142. Jiang T, Li M, Li Q, Guo Z, Sun X, Zhang X, et al. MicroRNA-98-5p inhibits cell proliferation and induces cell apoptosis in hepatocellular carcinoma via targeting IGF2BP1. Oncology research. 2017;25(7):1117.
143. Shen Q, Jiang S, Wu M, Zhang L, Su X, Zhao D. LncRNA HEIH confers cell sorafenib resistance in hepatocellular carcinoma by regulating miR-98-5p/PI3K/AKT pathway. Cancer Management and Research. 2020:6585-95.
144. Ji P-t, Wang X-y. Clinical application study on miR-98–5p as a prognostic biomarker in hepatocellular carcinoma. Clinics and Research in Hepatology and Gastroenterology. 2023;47(2):102077.
145. Fei X, Zhang P, Pan Y, Liu Y. MicroRNA-98-5p inhibits tumorigenesis of hepatitis B virus-related hepatocellular carcinoma by targeting NF-κB-inducing kinase. Yonsei Medical Journal. 2020;61(6):460.
146. Shi J, Ci Y, Zheng Y, Chen W, Chen X. Submicron silica particles have cytotoxicities on hepatocellular carcinoma, non-small cell lung cancer and breast cancer by unified regulating the XLOC_001659/miR-98-5p/MAP3K2-mediated pathway. Toxicology Research. 2021;10(4):824-34.
147. Li Y, Zhang Y, Zhang S, Huang D, Li B, Liang G, et al. circRNA circARNT2 suppressed the sensitivity of hepatocellular carcinoma cells to cisplatin by targeting the miR-155-5p/PDK1 axis. Molecular Therapy-Nucleic Acids. 2021;23:244-54.
148. Lin X-q, Huang Z-m, Chen X, Wu F, Wu W. XIST induced by JPX suppresses hepatocellular carcinoma by sponging miR-155-5p. Yonsei medical journal. 2018;59(7):816-26.
149. Fu X, Wen H, Jing L, Yang Y, Wang W, Liang X, et al. Micro RNA‐155‐5p promotes hepatocellular carcinoma progression by suppressing PTEN through the PI 3K/Akt pathway. Cancer science. 2017;108(4):620-31.
150. Eldosoky MA, Hammad R, Elmadbouly AA, Aglan RB, AbdelHamid SG, Alboraie M, et al. Diagnostic Significance of hsa-miR-21-5p, hsa-miR-192-5p, hsa-miR-155-5p, hsa-miR-199a-5p Panel and Ratios in Hepatocellular Carcinoma on Top of Liver Cirrhosis in HCV-Infected Patients. International Journal of Molecular Sciences. 2023;24(4):3157.
151. Wang J, Che J. CircTP63 promotes hepatocellular carcinoma progression by sponging miR-155-5p and upregulating ZBTB18. Cancer Cell International. 2021;21:1-13.

152. Chen G, Wang D, Zhao X, Cao J, Zhao Y, Wang F, et al. miR-155-5p modulates malignant behaviors of hepatocellular carcinoma by directly targeting CTHRC1 and indirectly regulating GSK-3β-involved Wnt/β-catenin signaling. Cancer cell international. 2017;17:1-12.
153. Atwa SM, Handoussa H, Hosny KM, Odenthal M, El Tayebi HM. Pivotal role of long non-coding ribonucleic acid-X-inactive specific transcript in regulating immune checkpoint programmed death ligand 1 through a shared pathway between miR-194-5p and miR-155-5p in hepatocellular carcinoma. World Journal of Hepatology. 2020;12(12):1211.
154. Ning S, Liu H, Gao B, Wei W, Yang A, Li J, et al. miR-155, miR-96 and miR-99a as potential diagnostic and prognostic tools for the clinical management of hepatocellular carcinoma. Oncology Letters. 2019;18(3):3381-7.
155. Liang F, Xu X, Tu Y. Resveratrol inhibited hepatocyte apoptosis and alleviated liver fibrosis through miR-190a-5p/HGF axis. Bioorganic & Medicinal Chemistry. 2022;57:116593.
156. Huang W, Chen Q, Dai J, Zhang Y, Yi Y, Wei X, et al. miR-744-5p suppresses tumor proliferation and metastasis by targeting transforming growth factor-beta 1 (TGF-β1) in hepatocellular carcinoma (HCC). Journal of Gastrointestinal Oncology. 2021;12(4):1811.
157. Li G, Tian Y, Gao Z. The role of AURKA/miR‐199b‐3p in hepatocellular carcinoma cells. Journal of Clinical Laboratory Analysis. 2022;36(12):e24758.
158. Cao M-Q, You A-B, Zhu X-D, Zhang W, Zhang Y-Y, Zhang S-Z, et al. miR-182-5p promotes hepatocellular carcinoma progression by repressing FOXO3a. Journal of hematology & oncology. 2018;11(1):1-12.
159. Wang S, Xu M, Sun Z, Yu X, Deng Y, Chang H. LINC01018 confers a novel tumor suppressor role in hepatocellular carcinoma through sponging microRNA-182-5p. American Journal of Physiology-Gastrointestinal and Liver Physiology. 2019;317(2):G116-G26.
160. Compagnoni C, Capelli R, Zelli V, Corrente A, Vecchiotti D, Flati I, et al. MiR-182-5p Is Upregulated in Hepatic Tissues from a Diet-Induced NAFLD/NASH/HCC C57BL/6J Mouse Model and Modulates Cyld and Foxo1 Expression. International Journal of Molecular Sciences. 2023;24(11):9239.
161. Zuo Z, Li Y, Zeng C, Xi Y, Tao H, Guo Y. Integrated analyses identify key molecules and reveal the potential mechanism of miR-182-5p/FOXO1 axis in alcoholic liver disease. Frontiers in Medicine. 2021;8:767584.
162. Su X, Su J, He H, Zhan Y, Liu H. Hsa_circ_0070269 inhibits hepatocellular carcinoma progression through modulating miR-182/NPTX1 axis. Biomedicine & Pharmacotherapy. 2019;120:109497.
163. Wei H, Wang J, Xu Z, Lu Y, Wu X, Zhuo C, et al. miR‐584‐5p regulates hepatocellular carcinoma cell migration and invasion through targeting KCNE2. Molecular Genetics & Genomic Medicine. 2019;7(6):e702.
164. Liu Q, Wang C, Jiang Z, Li S, Li F, Tan HB, et al. circRNA 001306 enhances hepatocellular carcinoma growth by up‐regulating CDK16 expression via sponging miR‐584‐5p. Journal of Cellular and Molecular Medicine. 2020;24(24):14306-15.
165. Liu Z, Lu J, Fang H, Sheng J, Cui M, Yang Y, et al. m6A modification-mediated DUXAP8 regulation of malignant phenotype and chemotherapy resistance of hepatocellular carcinoma through miR-584-5p/MAPK1/ERK pathway axis. Frontiers in Cell and Developmental Biology. 2021;9:783385.
166. Shao Z, Pan Q, Zhang Y. Hepatocellular carcinoma cell-derived extracellular vesicles encapsulated microRNA-584-5p facilitates angiogenesis through PCK1-mediated nuclear factor E2-related factor 2 signaling pathway. The International Journal of Biochemistry & Cell Biology. 2020;125:105789.
167. He Y, Huang H, Jin L, Zhang F, Zeng M, Wei L, et al. CircZNF609 enhances hepatocellular carcinoma cell proliferation, metastasis, and stemness by activating the Hedgehog pathway through the regulation of miR-15a-5p/15b-5p and GLI2 expressions. Cell death & disease. 2020;11(5):358.

168. Qiu R, Zeng Z. Hsa_circ_0006988 promotes sorafenib resistance of hepatocellular carcinoma by modulating IGF1 using miR-15a-5p. Canadian Journal of Gastroenterology and Hepatology. 2022;2022.
169. Zhang H-Y, Liang H-X, Wu S-H, Jiang H-Q, Wang Q, Yu Z-J. Overexpressed tumor suppressor exosomal miR-15a-5p in cancer cells inhibits PD1 expression in CD8+ T cells and suppresses the hepatocellular carcinoma progression. Frontiers in Oncology. 2021;11:622263.
170. Zhang Y, Tie Q, Bao Z, Shao Z, Zhang L. Inhibition of miR-15a-5p promotes the chemoresistance to pirarubicin in hepatocellular carcinoma via targeting eIF4E. Computational and Mathematical Methods in Medicine. 2021;2021.
171. Long J, Jiang C, Liu B, Fang S, Kuang M. MicroRNA-15a-5p suppresses cancer proliferation and division in human hepatocellular carcinoma by targeting BDNF. Tumor Biology. 2016;37:5821-8.
172. Li Y, Li D, Yang Y, Wang J. miR-15a-5p regulates liver cancer cell migration, apoptosis and cell cycle progression by targeting transcription factor E2F3. Critical Reviews™ in Eukaryotic Gene Expression. 2022;32(6).
173. Gao C, Wen Y, Jiang F, Gu X, Zhu X. Circular RNA circ_0008274 upregulates granulin to promote the progression of hepatocellular carcinoma via sponging microRNA-140-3p. Bioengineered. 2021;12(1):1890-901.
174. Li J, Zhao J, Wang H, Li X, Liu A, Qin Q, et al. MicroRNA-140-3p enhances the sensitivity of hepatocellular carcinoma cells to sorafenib by targeting pregnenolone X receptor. OncoTargets and therapy. 2018:5885-94.
175. Wen Z, Lian L, Ding H, Hu Y, Xiao Z, Xiong K, et al. LncRNA ANCR promotes hepatocellular carcinoma metastasis through upregulating HNRNPA1 expression. RNA biology. 2020;17(3):381-94.
176. Hou Z-H, Xu X-W, Fu X-Y, Zhou L-D, Liu S-P, Tan D-M. Long non-coding RNA MALAT1 promotes angiogenesis and immunosuppressive properties of HCC cells by sponging miR-140. American Journal of Physiology-Cell Physiology. 2020;318(3):C649-C63.
177. Xu X, Tao Y, Niu Y, Wang Z, Zhang C, Yu Y, et al. miR-125a-5p inhibits tumorigenesis in hepatocellular carcinoma. Aging (Albany NY). 2019;11(18):7639.
178. Kim JK, Noh JH, Jung KH, Eun JW, Bae HJ, Kim MG, et al. Sirtuin7 oncogenic potential in human hepatocellular carcinoma and its regulation by the tumor suppressors MiR‐125a‐5p and MiR‐125b. Hepatology. 2013;57(3):1055-67.
179. Xu J, Wang Y, Zhang Y, Dang S, He S. Astemizole promotes the anti-tumor effect of vitamin D through inhibiting miR-125a-5p-meidated regulation of VDR in HCC. Biomedicine & Pharmacotherapy. 2018;107:1682-91.
180. Zhang Y, Wu Q, Chang L, Liu J. miR-34a and miR-125a-5p inhibit proliferation and metastasis but induce apoptosis in hepatocellular carcinoma cells via repressing the MACC1-mediated PI3K/AKT/mTOR pathway. Neoplasma. 2020;67(5):1042-53.
181. Zheng J, Zhou Z, Xu Z, Li G, Dong P, Chen Z, et al. Serum microRNA-125a-5p, a useful biomarker in liver diseases, correlates with disease progression. Molecular medicine reports. 2015;12(1):1584-90.
182. Liu Y, Geng X. Long non-coding RNA (lncRNA) CYTOR promotes hepatocellular carcinoma proliferation by targeting the microRNA-125a-5p/LASP1 axis. Bioengineered. 2022;13(2):3666-79.
183. Li G, Zhang W, Gong L, Huang X. MicroRNA 125a-5p inhibits cell proliferation and induces apoptosis in hepatitis B virus-related hepatocellular carcinoma by downregulation of ErbB3. Oncology Research. 2019;27(4):449.
184. Aly DM, Gohar NA-H, Abd El-Hady AA, Khairy M, Abdullatif MM. Serum microRNA let-7a-1/let-7d/let-7f and miRNA 143/145 gene expression profiles as potential biomarkers in HCV induced hepatocellular carcinoma. Asian Pacific journal of cancer prevention: APJCP. 2020;21(2):555.

185. Infante‐Menéndez J, López‐Pastor AR, González‐Illanes T, González‐López P, Huertas‐Lárez R, Rey E, et al. Increased let‐7d‐5p in non‐alcoholic fatty liver promotes insulin resistance and is a potential blood biomarker for diagnosis. Liver International. 2023.
186. Fang F, Song T, Zhang T, Cui Y, Zhang G, Xiong Q. MiR-425-5p promotes invasion and metastasis of hepatocellular carcinoma cells through SCAI-mediated dysregulation of multiple signaling pathways. Oncotarget. 2017;8(19):31745.
187. Rao D, Guan S, Huang J, Chang Q, Duan S. miR-425-5p acts as a molecular marker and promoted proliferation, migration by targeting RNF11 in hepatocellular carcinoma. BioMed research international. 2020;2020.
188. Wu S, Liu S, Cao Y, Chao G, Wang P, Pan H. Downregulation of ZC3H13 by miR-362-3p/miR-425-5p is associated with a poor prognosis and adverse outcomes in hepatocellular carcinoma. Aging (Albany NY). 2022;14(5):2304.
189. Wu H, Shang J, Zhan W, Liu J, Ning H, Chen N. miR-425-5p promotes cell proliferation, migration and invasion by directly targeting FOXD3 in hepatocellular carcinoma cells. Molecular medicine reports. 2019;20(2):1883-92.
190. Wen-peng J, Wen-jing J, Xiu-juan G, Jun-hua F. Study on the mechanism of action of MiR-151a-3p in alcohol-related liver cancer. Journal of Hebei Medical University. 2021;42(11):1272.
191. Zhao S, Li J, Zhang G, Wang Q, Wu C, Zhang Q, et al. Exosomal miR-451a functions as a tumor suppressor in hepatocellular carcinoma by targeting LPIN1. Cell Physiol Biochem. 2019;53(1):19-35.
192. Wei G-Y, Hu M, Zhao L, Guo W-S. MiR-451a suppresses cell proliferation, metastasis and EMT via targeting YWHAZ in hepatocellular carcinoma. European Review for Medical & Pharmacological Sciences. 2019;23(12).
193. Xu Y, Lai Y, Cao L, Li Y, Chen G, Chen L, et al. Human umbilical cord mesenchymal stem cells-derived exosomal microRNA-451a represses epithelial–mesenchymal transition of hepatocellular carcinoma cells by inhibiting ADAM10. RNA biology. 2021;18(10):1408-23.
194. Zhao J, Li H, Zhao S, Wang E, Zhu J, Feng D, et al. Epigenetic silencing of miR-144/451a cluster contributes to HCC progression via paracrine HGF/MIF-mediated TAM remodeling. Molecular cancer. 2021;20(1):1-16.
195. Mu W, Gu P, Song W, Zhu T, Wang W, Zhou Y. Comprehensive analysis and identification of the circ_0084615/miR-451a/MEF2D axis in benzo (a) pyrene exposed tumor cells in hepato-carcinogenesis. Food and Chemical Toxicology. 2023;176:113810.
196. Lv C, Wan Q, Shen C, Wu H, Zhou B, Wang W. Long non-coding RNA ZSCAN16-AS1 promotes the malignant properties of hepatocellular carcinoma by decoying microRNA-451a and consequently increasing ATF2 expression. Molecular medicine reports. 2021;24(5):1-12.
197. Henry JC, Park J-K, Jiang J, Kim JH, Nagorney DM, Roberts LR, et al. miR-199a-3p targets CD44 and reduces proliferation of CD44 positive hepatocellular carcinoma cell lines. Biochemical and biophysical research communications. 2010;403(1):120-5.
198. Fornari F, Milazzo M, Chieco P, Negrini M, Calin GA, Grazi GL, et al. MiR-199a-3p regulates mTOR and c-Met to influence the doxorubicin sensitivity of human hepatocarcinoma cells. Cancer research. 2010;70(12):5184-93.
199. Yin J, Hou P, Wu Z, Wang T, Nie Y. Circulating miR-375 and miR-199a-3p as potential biomarkers for the diagnosis of hepatocellular carcinoma. Tumor Biology. 2015;36:4501-7.
200. Ren K, Li T, Zhang W, Ren J, Li Z, Wu G. miR-199a-3p inhibits cell proliferation and induces apoptosis by targeting YAP1, suppressing Jagged1-Notch signaling in human hepatocellular carcinoma. Journal of biomedical science. 2016;23:1-9.
201. Callegari E, D'Abundo L, Guerriero P, Simioni C, Elamin BK, Russo M, et al. miR-199a-3p modulates MTOR and PAK4 pathways and inhibits tumor growth in a hepatocellular carcinoma transgenic mouse model. Molecular Therapy-Nucleic Acids. 2018;11:485-93.

202. Liu W, Zhang R, Hou P, Wang J, Wu L, Li J. Circ-ZEB1 promotes PIK3CA expression by silencing miR-199a-3p and affects the proliferation and apoptosis of hepatocellular carcinoma. Molecular cancer. 2022;21(1):1-15.
203. Zhang Q, Cheng Q, Xia M, Huang X, He X, Liao J. Hypoxia-induced lncRNA-NEAT1 sustains the growth of hepatocellular carcinoma via regulation of miR-199a-3p/UCK2. Frontiers in oncology. 2020;10:998.
204. Kim JH, Badawi M, Park J-K, Jiang J, Mo X, Roberts LR, et al. Anti-invasion and anti-migration effects of miR-199a-3p in hepatocellular carcinoma are due in part to targeting CD151. International journal of oncology. 2016;49(5):2037-45.
205. Guan J, Liu Z, Xiao M, Hao F, Wang C, Chen Y, et al. MicroRNA-199a-3p inhibits tumorigenesis of hepatocellular carcinoma cells by targeting ZHX1/PUMA signal. American Journal of Translational Research. 2017;9(5):2457.
206. Li Z, Zhou Y, Zhang L, Jia K, Wang S, Wang M, et al. microRNA-199a-3p inhibits hepatic apoptosis and hepatocarcinogenesis by targeting PDCD4. Oncogenesis. 2020;9(10):95.
207. Ghosh A, Dasgupta D, Ghosh A, Roychoudhury S, Kumar D, Gorain M, et al. MiRNA199a-3p suppresses tumor growth, migration, invasion and angiogenesis in hepatocellular carcinoma by targeting VEGFA, VEGFR1, VEGFR2, HGF and MMP2. Cell death & disease. 2017;8(3):e2706-e.
